# Ten weeks of branched chain amino acid supplementation improves select performance and immunological variables in trained cyclists

**DOI:** 10.1186/1550-2783-12-S1-P20

**Published:** 2015-09-21

**Authors:** Wesley C Kephart, Taylor D Wachs, R Mac Thompson, C Brooks Mobley, Carlton D Fox, James R McDonald, Brian S Ferguson, Kaelin C Young, Ben Nie, Jeffrey S Martin, Joseph M Company, David D Pascoe, Robert D Arnold, Jordan R Moon, Michael D Roberts

**Affiliations:** 1 School of Kinesiology, Auburn University, Auburn, AL, USA; 2 Edward Via College of Osteopathic Medicine, Auburn Campus, Auburn, AL, USA; 3 Harrison School of Pharmacy, Auburn University, Auburn, AL, USA; 4 Endurance Company, LLC, Bloomington, IL, USA; 5 MusclePharm Sports Science Institute, Denver, CO, USA

## Background

We examined if supplementing trained cyclists (32 ± 2 yr, 77.8 ± 2.6 kg, and 7.4 ± 1.2 yr training) with 12g/d (6g/d L-Leucine, 2g/d L-Isoleucine and 4g/d L-Valine) of either branched chain amino acids (BCAAs, n = 9) or a maltodextrin placebo (PLA, n = 9) over a 10-week training season affected select body composition, performance, and/or immune variables.

## Methods

Before and after the 10-week study, the following was assessed: a) 4-h fasting blood draws; b) dual X-ray absorptiometry body composition; c) Wingate peak power tests; and d) 4km time-trials.

## Results

No group*time interactions existed for total lean mass (p = 0.27) or dual-leg lean mass (p = 0.96). A significant interaction existed for body mass-normalized relative peak power (19% increase in the BCAA group pre- to post-study, p = 0.01), and relative mean power (4% increase in the BCAA group pre- to post-study, p = 0.01). 4km time-trial time to completion approached a significant interaction (p = 0.08), as the BCAA group improved in this measure by 11% pre- to post-study, though this was not significant (p = 0.15). There was a tendency for the BCAA group to present a greater post-study serum BCAA: L-Tryptophan ratio compared to the PLA group (p = 0.08). A significant interaction for neutrophil number existed (p = 0.04), as there was a significant 18% increase within the PLA group from the pre- to post-study time point (p = 0.01).

## Conclusions

Chronic BCAA supplementation improves sprint performance variables in endurance cyclists. Additionally, given that BCAA supplementation blunted the neutrophil response to intense cycling training, BCAAs may benefit immune function during a prolonged cycling season.

